# *IGFBP2* expression predicts IDH-mutant glioma patient survival

**DOI:** 10.18632/oncotarget.13329

**Published:** 2016-11-12

**Authors:** Lin Eric Huang, Adam L. Cohen, Howard Colman, Randy L. Jensen, Daniel W. Fults, William T. Couldwell

**Affiliations:** ^1^ Department of Neurosurgery, Clinical Neurosciences Center, Salt Lake City, Utah, USA; ^2^ Department of Oncological Sciences, Huntsman Cancer Institute, Salt Lake City, Utah, USA; ^3^ Division of Oncology, Huntsman Cancer Institute, Salt Lake City, Utah, USA

**Keywords:** DNA hypermethylation, glioma, IDH, IGFBP2, prognosis

## Abstract

Mutations of the isocitrate dehydrogenase (IDH) 1 and 2 genes occur in ~80% of lower-grade (WHO grade II and grade III) gliomas. Mutant IDH produces (*R*)-2-hydroxyglutarate, which induces DNA hypermethylation and presumably drives tumorigenesis. Interestingly, IDH mutations are associated with improved survival in glioma patients, but the underlying mechanism for the difference in survival remains unclear. Through comparative analyses of 286 cases of IDH-wildtype and IDH-mutant lower-grade glioma from a TCGA data set, we report that IDH-mutant gliomas have increased expression of tumor-suppressor genes (*NF1*, *PTEN,* and *PIK3R1*) and decreased expression of oncogenes(*AKT2*, *ARAF*, *ERBB2*, *FGFR3*, and *PDGFRB*) and glioma progression genes (*FOXM1*, *IGFBP2*, and *WWTR1*) compared with IDH-wildtype gliomas. Furthermore, each of these genes is prognostic in overall gliomas; however, within the IDH-mutant group, none remains prognostic except *IGFBP2* (encodinginsulin-like growth factor binding protein 2). Through validation in an independent cohort, we show that patients with low *IGFBP2* expressiondisplay a clear advantage in overall and disease-free survival, whereas those with high *IGFBP2* expressionhave worse median survival than IDH-wildtype patients. These observations hold true across different histological and molecular subtypes of lower-grade glioma. We propose therefore that an unexpected biological consequence of IDH mutations in glioma is to ameliorate patient survival by promoting tumor-suppressor signaling while inhibiting that of oncogenes, particularly *IGFBP2*.

## INTRODUCTION

Malignant gliomas are the most common primary brain tumors in adults [1], yet recurrent genetic changes that drive pathogenesis of WHO grade II and grade III gliomas (referred to collectively hereafter as lower-grade glioma, LGG) remain to be identified [2, 3]. Single somatic mutations of the isocitrate dehydrogenase 1 (*IDH1)* gene, predominantly R132H, occurred in ~80% of LGGs as well as in secondary glioblastomas [4, 5]. *IDH1* encodes the cytosolic isocitrate dehydrogenase 1, an enzyme that catalyzes the conversion of isocitrate to 2-oxoglutarate (aka α-ketoglutarate) concomitant with the production of reduced nicotinamide adenine dinucleotide phosphate. Tumors lacking mutations in *IDH1* often have single mutations in *IDH2*, a mitochondrial gene in the citric acid cycle.

The most striking biochemical finding from *IDH1* and *IDH2* mutations is the acquired neomorphic activity to catalyze the reduction of 2-oxoglutarate to the (*R*)-enantiomer of 2-hydroxyglutarate [(*R*)^2^-HG] [6, 7]. (*R*)2-HG has been shown to be a competitive inhibitor of multiple 2-oxoglutarate-dependent dioxygenases, including histone demethylases and the TET family of 5-methylcytosine hydroxylases [8, 9]. In keeping with this, mutant IDH1 inhibits histone demethylation and induces DNA hypermethylation in cell culture and animal model, thereby blocking cell differentiation [10–12]. Likewise, glioblastomas and LGGs harboring IDH mutations manifest a CpG island methylator phenotype [13, 14]. Although pharmacological targeting of mutant IDH1 or IDH2 induces tumor cell differentiation through reduction of (*R*)2-HG production [15, 16], the inhibitory effect on glioma growth remains less clear [16, 17], and there are no appreciable changes in genome-wide DNA methylation [16]. Therefore, the role of DNA hypermethylation and its target gene regulation remain obscure in glioma biology. An intriguing clinical feature of IDH-mutant glioma is the prolonged overall survival in comparison with the IDH-wildtype: ranging from a two- to threefold increase in patients with glioblastomas to a three- to fivefold increase in those with LGGs [2, 4, 5]. Whether DNA methylation-associated gene expression is a cause of the survival advantage has yet to be explored.

To address these questions, we performed comparative analyses of 286 cases of IDH-wildtype and IDH-mutant LGGs to identify differential expression of genes that not only account for prolonged survival but also correlate specifically with IDH mutations.

## RESULTS

### Increased expression of *NF1* and *PTEN* in IDH-mutant gliomas correlates with improved patient survival

**Figure 1:**
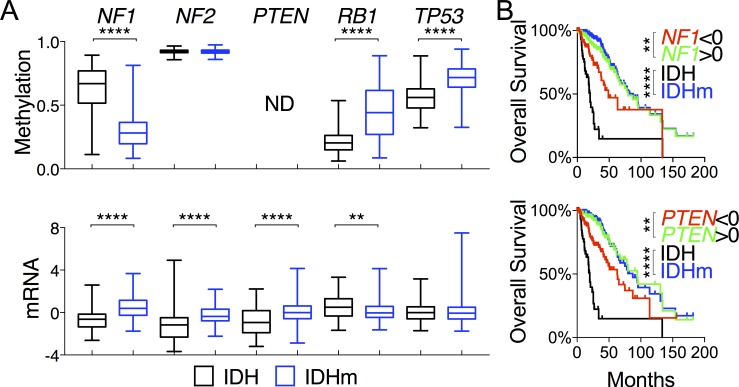
Increased *NF1* and *PTEN* expression in IDH-mutant glioma correlates with overall survival of glioma patients **A.** DNA methylation and mRNA (in z-scores) of tumor-suppressor genes are presented in box-and-whisker plots. Unpaired *t*-tests were performed to compare IDH-wildtype (IDH) and IDH-mutant (IDHm) gliomas. **B.** Overall survival with respect to mRNA levels of specified genes, superimposed with the survival curves of IDH-wildtype and IDH-mutant gliomas, is presented with p values in log-rank (Mantel-Cox) tests. Decreased (< 0 in z-scores) and increased (> 0 in z-scores) mRNA levels were used for comparison. **, *p* < 0.01; ****, *p* < 0.0001; ND, not done.

**Figure 2:**
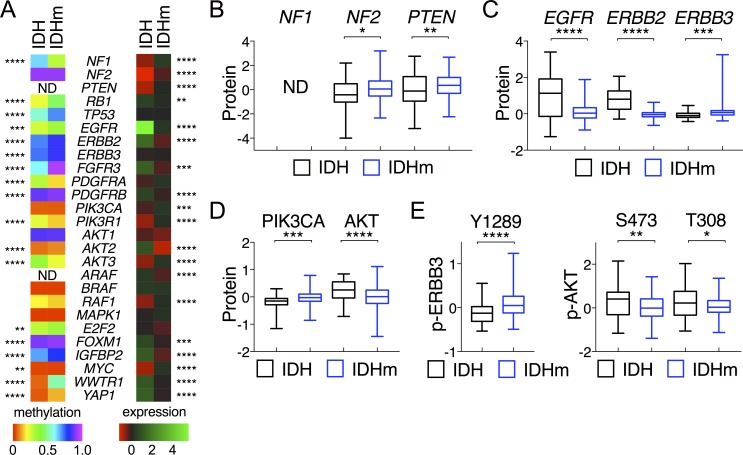
Comparative analysis of DNA methylation and gene expression between IDH-wildtype and IDH-mutant gliomas **A.** Heat maps of mean DNA methylation values (left) and mean mRNA levels (right) of corresponding genes are presented with statistical significance marked between IDH and IDHm groups. **B.**-**D.** Protein abundance (in z-scores) of specified genes are presented in box-and-whisker plots. **E.** Phosphorylation of ERBB3 (p-ERBB3) at Y1289 and AKT (p-AKT) at S473 and T308 are presented in the same way as above. Unpaired *t*-tests were performed for comparison between IDH and IDHm groups. *, *p* < 0.05; ***, *p* < 0.001.

To provide an explanation for the improved survival in IDH-mutant patients relative to the wildtype patients, we first focused on glioma-relevant genes that possess tumor-suppressing activities, one of the key hallmarks of cancer [18, 19]. We compared the methylation and expression data between IDH-wildtype and IDH-mutant gliomas. Surprisingly, the IDH-mutant group exhibited notably decreased *NF1* methylation concomitant with increased mRNA levels in reference to the wild type (Figures 1A and 2A). The mean mRNA and protein levels of *NF2* and *PTEN* were also much higher than those in IDH-wildtype gliomas (Figure 2B). It should be noted that mutations of *NF1* and *PTEN* were almost exclusive to IDH-wildtype glioma [2] (Table S1), indicating the functional importance of increased *NF1* and *PTEN* expression in IDH-mutant glioma. Although IDH-mutant gliomas had lower *RB1* expression along with higher methylation, the expression of RB pathway genes *CDKN2A* and *CDKN2B* was maintained (Figure S1A). Similarly, increased *TP53* methylation in the IDH-mutant group did not seem to affect the gene expression, and, furthermore, genes that negatively regulate *TP53* (i.e., *MDM1, MDM2,* and *MDM4*) were all expressed at significantly lower levels in IDH-mutant glioma than in IDH-wildtype glioma. Even though the roles of *RB1* and *TP53* seem less clear, the results nevertheless suggest that despite an overall increase in DNA methylation, IDH-mutant gliomas display increased expression of tumor-suppressor genes *NF1*, *NF2*, and *PTEN*.

To assess the importance of these genes in LGG, we asked whether the expression levels correlate with patient survival, and if so, whether such correlation is relevant within the IDH-mutant group. IDH-mutant patients had a median overall survival of 87 months versus 20 months in IDH-wildtype patients (Table S2). By comparison, patients with high expression of *NF1* and *PTEN* showed a similar survival advantage, whereas patients with low expression of these genes were still better off than those of IDH-wildtype (Figure 1B; Table S2). When analyzed within IDH-mutant gliomas, however, *NF1* and *PTEN* expression did not confer a survival advantage (Figure S1B), suggesting that their correlation with improved survival is associated with IDH mutation-mediated increase in gene expression. Although no survival advantage was observed with respect to *NF2* expression (data not shown), low expression of *RB1*, unexpectedly, showed a modest correlation with improved survival (Table S2). Additionally, none of the putative tumor-suppressor genes relevant to LGG, namely, *ATRX*, *CIC*, *FUBP1*, and *NOTCH1* [2], showed correlations with overall survival (data not shown). Taken together, these results indicate that the increased *NF1* and *PTEN* expression in IDH-mutant gliomas may be partly responsible for the improved patient survival compared with IDH-wildtype gliomas.

### Repressed RTK-PI3K-AKT signaling in IDH-mutant glioma

Because sustaining proliferative signaling is another hallmark of cancer [18], we explored the receptor tyrosine kinase (RTK) signal pathways by analyzing genes known to promote the EGFR, FGFR, and PDGFR signaling commonly seen in malignant gliomas [2, 20]. We observed increased DNA methylation in *EGFR*, *ERBB2*, *ERBB3*, *FGFR3*, and *PDGFRB* in IDH-mutant gliomas as the mean methylation of each gene was statistically greater than that of IDH-wildtype (Figure 2A; Figure S2A). Concomitantly, the mean *EGFR*, *ERBB2*, *FGFR3*, and *PDGFRB* mRNA levels were statistically lower than those of IDH-wildtype gliomas, which also exhibited a marked increase in EGFR and ERBB2 protein abundance (Figure 2C). Consistent with the recent report of more chromosome gains in regions containing RTK pathways in IDH-wildtype gliomas [21], this finding suggests a weakened RTK signaling in IDH-mutant glioma compared with IDH-wildtype glioma, as suggested previously [2].

Despite increased DNA hypermethylation, the mean ERBB3 abundance and phosphorylation were statistically increased in IDH-mutant gliomas (Figure 2C and 2E), as shown previously [2]. Furthermore, the mean *PDGFRA* methylation was unexpectedly decreased in IDH-mutant glioma, but the mRNA levels had a modest, yet statistically insignificant, increase (Figure S2A), similar to that noted previously [22].

With respect to the downstream components of the phosphoinositol 3-kinase (PI3K)–AKT pathway, the mean mRNA levels of *PIK3R1* (encoding the PI3K regulatory subunit 1) were much higher than those of IDH-wildtype, whereas the mean mRNA levels of *AKT2* were statistically lower, consistent with the opposite directional changes in DNA methylation (Figure 2A; Figure S2B). Despite the increased *PIK3CA* (encoding the PI3K catalytic subunit α) and *AKT3* expression in IDH-mutant glioma, the overall AKT activities were statistically lower than those of IDH-wildtype, as indicated by the mean AKT phosphorylation at Ser-473 and Thr-308 (Figure 2D and 2E). Taken together, these results indicate that IDH-mutant gliomas exhibit repressed RTK-PI3K-ATK signaling in addition to increased expression of tumor-suppressor genes.

### Increased expression of *PIK3R1* and *ERBB3* and decreased expression of *ERBB2, FGFR3,* and *AKT2* correlate with improved patient survival

**Figure 3:**
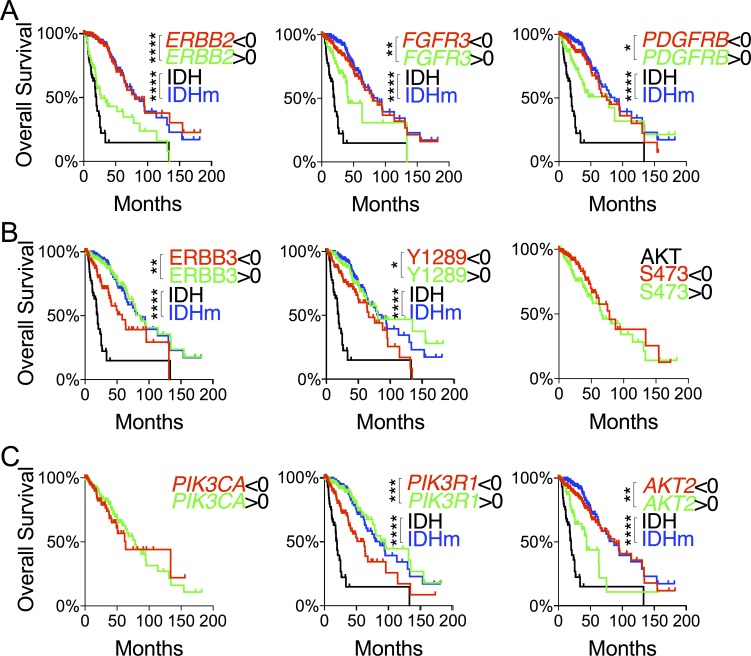
Evaluation of differentially expressed genes in the RTK-PI3K-AKT pathway with respect to glioma patient survival **A.** and **C.** Overall survival with respect to mRNA levels of RTK genes **A.** and PI3K-AKT genes **C.** that were differentially expressed between IDH and IDHm groups. **B.** Overall survival with respect to ERBB3 abundance and ERBB3 and AKT phosphorylation. Decreased (< 0 in z-scores) and increased (> 0 in z-scores) protein abundance and phosphorylation were used for log-rank (Mantel-Cox) tests and presented with p values (unless not significant). The survival curves with statistical significance were compared with those of IDH-wildtype and IDH-mutant gliomas.

To assess the functional relevance of the repressed RTK-PI3K-AKT signaling in patient survival, we focused on genes differentially expressed as described above. No statistical survival advantage was observed with *EGFR* nor with *PDGFRA* transcripts despite the distinct differences at mRNA and protein levels between IDH-wildtype and IDH-mutant gliomas (Figure S3A). Of note, high expression of *PDGFRA* seemed rather associated, if at all, with improved overall survival. However, low expression of *ERBB2* correlated statistically with improved overall and disease-free survival (Figure 3A; Figure S4A). Likewise, low expression of *FGFR3* and *PDGFRB* also correlated with overall but not disease-free survival. Unexpectedly, high ERBB3 abundance and Tyr-1289 phosphorylation correlated with improved overall and disease-free survival (Figure 3B; Figure S3B).

High expression of *PIK3R1* and low expression of *AKT2* correlated statistically with improved overall and disease-free survival (Figure 3C; Figure S4B). By contrast, no survival benefits were observed with respect to *PIK3CA* expression and AKT phosphorylation at Ser-473 and Thr-308 (Figure 3B; Figure S3C). It is noteworthy that the median survival of IDH-wildtype patients remained the worst regardless of the expression levels of RTK-PI3K-AKT genes. Finally, within IDH-mutant gliomas, we found no specific predictive values for any of these genes (Figure S5). Therefore, we conclude that increased ERBB3 abundance and *PIK3R1* expression and decreased *ERBB2*, *FGFR3*, and *AKT2* expression may explain the improved LGG patient survival in IDH-mutant gliomas compared with IDH-wildtype gliomas.

### Suppression of *ARAF* in IDH-mutant glioma correlates with improved survival

Also implicated in LGG pathogenesis is the BRAF-MAP kinase pathway [2], but no significant changes in *RAF1*, *BRAF*, and *MAPK1* methylation were observed in IDH-mutant glioma in comparison with IDH-wildtype (Figure S6A). Differential expression was found, however, with increased mean *RAF1* transcripts and MAPK1 abundance and decreased mean *ARAF* transcripts in IDH-mutant gliomas; yet only low *ARAF* expression correlated with improved patient survival (Figure S6B).

### *IGFBP2* expression inversely correlates with overall survival of IDH-mutant glioma patients

**Figure 4:**
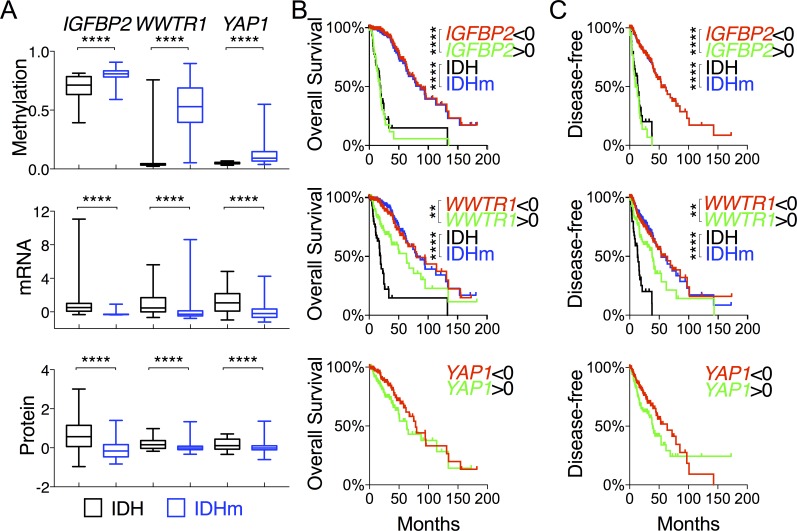
Comparative and survival analyses of glioma progression genes between IDH-wildtype and IDH-mutant gliomas **A.** DNA methylation, mRNA (in z-scores), and protein abundance (in z-scores) of genes, as indicated, are presented in box-and-whisker plots. **B.** and **C.** Overall survival **B.** and disease-free survival **C.** with respect to the mRNA levels of specified genes were analyzed in log-rank (Mantel-Cox) tests and presented with p values (unless not significant). The survival curves with statistical significance were compared with those of IDH-wildtype and IDH-mutant gliomas.

**Figure 5:**
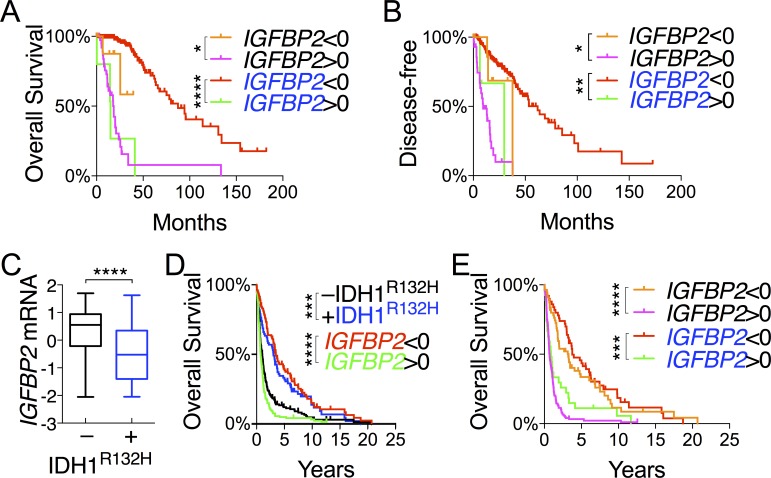
*IGFBP2* expression is a validated prognostic marker within IDH-mutant glioma **A.** and **B.** Correlations of *IGFBP2* mRNA levels with overall survival **A.** and disease-free survival **B.** of IDH-wildtype patients (in black) and IDH-mutant patients (in blue) were analyzed in log-rank (Mantel-Cox) tests. **C.**-**E.** The GSE16011 glioma data set was used for validation of *IGFBP2* as a prognostic marker in gene expression between IDH1^R132H^-negative and IDH1^R132H^-positive gliomas **C.**, and overall survival of all glioma patients **D.** and patients stratified based on IDH status **E.** with respect to *IGFBP2* expression. IDH1^R132H^-negative is in black, and IDH1^R132H^-positive in blue.

In a continued effort to identify genes that are specifically associated with the survival of IDH-mutant gliomas, we examined *IGFBP2*, *WWTR1*, and *YAP1*, which are associated with glioma progression [23–26]. As expected, IDH-mutant gliomas manifested increased methylation concomitant with decreased expression of *IGFBP2*, *YAP1,* and *WWTR1* in comparison with IDH-wildtype (Figures 2A and 4A). The mean mRNA and protein levels of the three genes were statistically much lower in IDH-mutant gliomas. Patients with low expression of *IGFBP2* and *WWTR* but not *YAP1* had essentially the same median overall and disease-free survival as IDH-mutant patients (Figure 4B and 4C; Tables S2 and S3). Similar survival advantages were seen with analysis of IGFBP2 but not WWTR1 protein abundance (Figure S7A and S7B). It is noteworthy, however, that patients with high *IGFBP2* mRNA levels were the only group that showed worse survival than IDH-wildtype patients. Furthermore, within the IDH-mutant group, *IGFBP2* was the only gene that was still prognostic at the mRNA levels; the median overall survival of IDH-mutant gliomas with high *IGFBP2* expression was ~25% worse than that of IDH-wildtype (Figure 5A and 5B; Table S2). Further analysis of IGFBP2 protein abundance confirmed the prognostic value in overall survival within the IDH-mutant group (Figure S7C).

To test further the specificity of *IGFBP2*, we also analyzed *MYC*, *FOXM1*, and *E2F2,* which have been associated with malignant progression of IDH-mutant glioma [3]. Interestingly, IDH-mutant gliomas showed statistically lower *FOXM1* but higher *MYC* mRNA levels, which nevertheless correlated with improved overall survival similar to that of IDH mutations (Figure 2A; Figure S8A), and yet the IDH-wildtype group remained the worst in survival regardless of the expression levels of these genes. Furthermore, within the IDH-mutant group, no survival benefits were observed with statistical significance (Figure S8B).

To validate our finding of *IGFBP2* as a specific prognostic marker of IDH-mutant glioma, we used an independent cohort of gliomas ranging from WHO grade II to grade IV with IDH status determined by immunohistochemistry against IDH1^R132H^ [27]. Again, not only were *IGFBP2* mRNA levels statistically much lower in the IDH1^R132H^-positive than in the IDH1^R132H^-negative group, but low *IGFBP2* mRNA levels also correlated with improved survival whereas high *IGFBP2* mRNA levels exhibited worse survival than the IDH1^R132H^-negative group (Figure 5C and 5D). Importantly, the survival benefit of low *IGFBP2* expression held true within the IDH1^R132H^-positive group (Figure 5E). Taken together, these results indicate that IDH-mutant glioma patients generally manifest low *IGFBP2* expression, which is associated with improved survival independent of IDH status, whereas high *IGFBP2* expression results in worse survival than in the IDH-wildtype group.

### Across all histological types *IGFBP2* expression inversely correlates with overall survival

**Figure 6:**
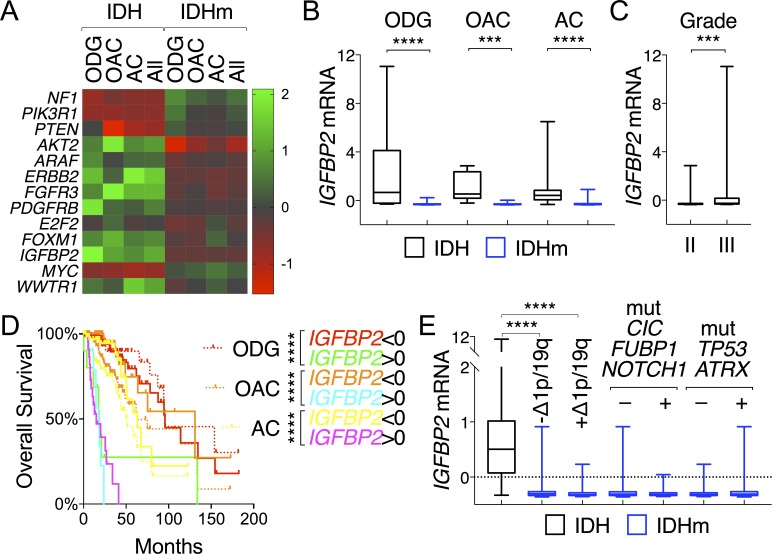
*IGFBP2* is prognostic across all histological types of glioma **A.** A heat map depicts distinctive patterns of gene expression between IDH-wildtype and IDH-mutant gliomas (All) but a shared pattern among oligodendroglioma (ODG), oligoastrocytoma (OAC), and astrocytoma (AC) of the same IDH status. The listed genes were all associated with glioma patient survival. **B.** and **C.** *IGFBP2* mRNA levels were compared between IDH and IDHm groups in each histological type **B.** and between tumor grades **C.**. **D.** Differential *IGFBP2* expression correlated with distinct patterns of survival in all histological types in reference to their respective survival curves (in dotted lines). **E.** Within IDH-mutant gliomas, there was no statistical difference in mean *IGFBP2* expression between subtypes with and without 1p/19q deletions, and between gliomas with and without mutations in *CIC, FUBP1,* and/or *NOTCH1* and between those with and without mutations in *TP53* and/or *ATRX*.

LGGs have been classified histologically into oligodendroglioma, oligoastrocytoma, and astrocytoma subtypes [28], with oligodendroglioma demonstrating the best overall survival and astrocytoma having the worst [2]. To test the possibility that the above-described gene expression is associated with specific LGG histological types and/or grades, we evaluated the expression pattern of tumor-suppressor genes (*NF1*, *PIK3R1*, and *PTEN*), oncogenes (*AKT2*, *ARAF*, *ERBB2*, *FGFR3*, and *PDGFRB*), and progression genes (*E2F2*, *FOXM1*, *IGFBP2*, *MYC*, and *WWTR1*) that had been shown above to be correlative with patient survival. By using a heat map that corresponded to the histological types and IDH status (Figure 6A), we observed that irrespective of the histological types, 1) the IDH-mutant group expressed higher levels of tumor-suppressor genes than the IDH-wildtype group albeit with various degrees of statistical significance (Figure S9); 2) by contrast, the latter expressed higher levels of oncogenes, apparently with gene-specific variations among different histological types; and 3) the expression of progression genes was generally higher in the IDH-wildtype group, with the exception of *MYC*, where high expression correlated with improved survival in IDH-mutant patients (Figure S8). Altogether, these findings provide molecular explanations for the survival advantage of IDH-mutant patients in all histological types.

Furthermore, differential *IGFBP2* expression between IDH-wildtype and IDH-mutant gliomas was seen with strong statistical significance across all three histological types, and increased *IGFBP2* expression correlated with higher grade (Figure 6B and 6C), a finding that supports the general role of *IGFBP2* in glioma progression. Consistently, differential *IGFBP2* expression in each histological type was prognostic with strong statistical significance; decreased expression extended median survival by 24% in astrocytoma and 106% in oligoastrocytoma, whereas increased expression shortened median survival by >70% for astrocytoma and oligoastrocytoma and >80% for oligodendroglioma (Figure 6D). The latest classification of IDH-mutant glioma groups into two subtypes: one with 1p/19q co-deletion also harboring mutations in *CIC*, *FUBP1*, and *NOTCH1*, and the other without 1p/19q co-deletion but with extreme high frequencies of mutations in *TP53* and *ATRX* [2,29]. When IDH-mutant gliomas were stratified accordingly with 1p/19q co-deletion, we found no statistical differences in *IGFBP2* expression between the two subtypes even though sharp differences remained between IDH-wildtype group and each subtype (Figure 6E). Furthermore, no statistical differences in *IGFBP2* expression were observed between subtypes with and without *CIC*, *FUBP1*, and/or *NOTCH1* mutations, nor between those with and without *TP53* and/or *ATRX* mutations, which further disputes the possibility of *IGFBP2* association with specific glioma subtypes. Taken together, these results indicate that although *IGFBP2* expression is low in IDH-mutant gliomas of all three histological types, the increased expression is a powerful, negative prognostic marker that predicts a median overall survival worse than that of IDH-wildtype group.

## DISCUSSION

The suggestion that *IGFBP2* is involved in glioma progression dates back to 1999 when *IGFBP2* overexpression was discovered in glioblastoma [30]. Further studies demonstrated the important role of IGFBP2 in glioma progression [23, 24] and indicated that *IGFBP2* expression was a poor prognostic marker in a mixed cohort ranging from grade II glioma to glioblastoma [31] as well as in glioblastoma alone [32]. Interestingly, our analysis of 135 cases of TCGA glioblastoma data also showed a 2-month statistical advantage in the median overall survival with low *IGFBP2* mRNA levels (data not shown). However, this advantage reduced by half and lost statistical significance when the only 7 IDH-mutant cases were subtracted from the analysis, further supporting an intimate relationship between IDH mutations and *IGFBP2* in glioblastoma prognosis.

To our knowledge, *IGFBP2* is the first gene that serves as a prognostic marker in IDH-mutant glioma patients; in particular, increased *IGFBP2* expression in IDH-mutant glioma patients is prognostic of poor outcomes worse than those of IDH-wildtype glioma patients. Although many genes identified in this study may serve as prognostic markers of lower-grade gliomas, only *IGFBP2* remains prognostic within IDH-mutant glioma patients. Low *IGFBP2* expression seems attributable to global DNA hypermethylation [33], but the regulatory mechanism seems complex with the possible involvement of copy number (data not shown); thus, further investigations are warranted to understand how *IGFBP2* becomes deregulated to drive glioma progression.

The key hallmarks of cancer involve sustaining proliferative, replicative, and angiogenic signaling while evading tumor-suppressive and cell-death signaling [18]. We demonstrate here the strong statistical correlations of IDH-mutant gliomas with increased expression of mostly intact tumor-suppressor genes (*NF1, PIK3R1,* and *PTEN*), and decreased expression of oncogenic genes (*ERBB2*, *FGFR3*, *PDGFRB*, *AKT2,* and *ARAF*) and progression genes (*IGFBP2, FOXM1,* and *WWTR1*). Furthermore, these changes in gene expression are associated statistically with improved patient survival. Additionally, we observed decreased replicative potential and reduced apoptotic and hypoxic/angiogenic signaling (data not shown) in IDH-mutant gliomas. Therefore, these results not only provide molecular explanation to the mystery of prolonged survival of IDH-mutant glioma patients but also prompt us to revisit the functional role of IDH mutations in glioma.

Acquisition of somatic mutations in the genome is an evolutionary process of cancer [34]. Whereas mutations that are causally implicated in oncogenesis are defined as driver mutations, the rest are grouped as passenger mutations for the lack of clear contributions to cancer development. Although IDH mutations are believed to drive glioma initiation by producing (*R*)2-HG, which results in DNA hypermethylation and metabolic reprograming [35–37], expression of mutant *Idh1* in the mouse brain has yet to induce glioma formation despite robust production of (*R*)2-HG [11, 38]. To the contrary, our results show strong correlation between increased DNA methylation and suppression of oncogenic signaling, suggesting that the effects of DNA hypermethylation in IDH-mutant glioma are unexpectedly anti-oncogenic, which is consistent with a newly identified subtype of IDH-mutant glioma featuring DNA demethylation associated with poor survival [39]. Our hypothesis is also supported by the strong correlations between IDH mutations and increased tumor-suppressor gene expression that correlates with patient survival. Likewise, targets of epigenetic silencing identified in IDH1-mutant gliomas, including glycolytic genes [40], retinol binding protein 1 [41], and microRNA miR148a [42], were all associated with reduced cell proliferation and improved survival. Furthermore, introduction of mutant IDH1 in glioblastoma cells and transformed astrocytes inhibited cell proliferation, reduced tumor growth, and improved mouse survival [43–45] (Tiburcio *et al*., manuscript in preparation). Thus, taken together, the evidence indicates that IDH mutations in glioma may represent a different category, i.e., beneficial mutations, resulting from an aberrant cellular response that counteracts glioma progression, even though the mechanism by which transformed cells acquire this type of mutations necessitates further investigation.

A counterargument to the theory of IDH mutation-associated survival advantage is that IDH-wildtype glioma is simply a different type of disease with more genetic abnormalities and therefore more malignant traits than IDH-mutant glioma. Given the fact that IDH mutations are early events (“founder mutation”) in LGG development [46], it stands to reason that the early gain of IDH mutations prevents glioma from acquiring additional malignant traits seen in IDH-wildtype glioma, whereas functional loss of IDH mutations, such as the loss of DNA hypermethylation in the newly identified IDH-mutant glioma subtype [39], results in unfavorable clinical outcome. In fact, the propensity of IDH-mutant tumor cells to lose the mutant allele and (*R*)2-HG production has been observed in culture [40] even though whether this occurs *in vivo* merits further investigation.

This study has also prompted us to reassess therapeutic targets of malignant glioma. Although pharmacological targeting of IDH mutations induces tumor cell differentiation by reducing (*R*)2-HG production [15,16], the inhibitory effect on glioma growth remains less clear [16,17]. Additionally, a recent study suggests that IDH1-mutant inhibitors may alter oxidative stress responses in glioma patients and therefore diminish the therapeutic efficacy of irradiation [47]. Our results not only suggest that targeting of mutant IDH in glioma patients could be counterproductive but also raise concerns about the selection of molecular targets for malignant glioma treatment. For instance, owing to the lack of correlation between overall survival and expression of *EGFR* or *PDGFRA*, targeting the corresponding pathway may not be effective, even in tumors overexpressing *EGFR* or *PDGFRA*. By contrast, targeting ERBB3 signaling could be counterproductive given the statistical association of ERBB3 abundance and activity with improved survival. In sum, deep understanding of the signaling pathway critical for patient survival may serve as the foundation for improving therapeutic efficacy of malignant glioma.

## MATERIALS AND METHODS

### Glioma data sets

Genomic data and patient survival data from the Brain Lower Grade Glioma (TCGA, Provisional) data set with 286 cases of sequenced tumors were acquired from cBioPortal (http://www.cbioportal.org/) [48,49]. They include mutation data from whole-exome sequencing, mRNA expression z-scores (RNA Seq V2 RSEM), which compared with the gene distribution of each gene in tumors that are diploid for this gene, DNA methylation (HM450) beta-values, and protein expression and phosphorylation results by reverse-phase protein array. The genomic data along with glioma grades and histologic types were matched with patient survival data according to the case identification numbers. There were 53 cases of IDH-wildtype gliomas and 233 cases with either *IDH1* or *IDH2* mutations, which were grouped into a single group, IDH-mutant. The GSE16011 cohort of gliomas ranging from WHO grade II to grade IV was used for validation [27], wherein the IDH status was determined by immunohistochemistry against IDH1^R132H^.

### Comparative analysis and generation of heat maps

We used the GraphPad Prism software to perform column analyses where unpaired *t*-tests were used to compare the differences between IDH-wildtype gliomas and IDH-mutant gliomas with respect to methylation, mRNA, protein, and phosphorylation of individual genes. The results were presented in box-and-whisker plots. Two-tailed *p* values were used for statistical significance (*, *p*<0.05; **, *p*<0.01; ***, *p*<0.001; and ****, *p*<0.0001). Heat maps of DNA methylation and gene expression were generated using the mean of the genomic data and plotted accordingly.

### Survival study

Log-rank (Mantel-Cox) tests were performed to compare gene expression *z*-scores below zero and above zero in relation to overall survival or disease-free survival data. Median overall survival and disease-free survival are listed in Table S2 and Table S3 along with log-rank hazard ratio and 95% confidence interval of ratio. Kaplan-Meier survival curves were plotted with censored points marked. For overall survival analysis, there were 53 IDH-wildtype cases and 229 IDH-mutant cases available. For disease-free survival analysis, 42 IDH-wildtype and 218 IDH-mutant cases were available.

## SUPPLEMENTARY MATERIAL





## Abbreviations

IDH: isocitrate dehydrogenase
IDHm: IDH-mutant
IGFBP2: insulin-like growth factor binding protein 2
LGG: lower-grade glioma
PI3K: phosphoinositol 3-kinase
RTK: receptor tyrosine kinase
TCGA: The Cancer Genome Atlas
WHO: World Health Organization.
