# Supplementary material for: IGFBP2 expression predicts IDH-mutant glioma patient survival
Source: Oncotarget. 2016 Nov 12;8(1):191–202. doi: 10.18632/oncotarget.13329 (PMC5352106; doi:10.18632/oncotarget.13329)
Supplement: Supplementary file 1 [file oncotarget-08-191-s001.pdf]

IGFBP2 expression predicts IDH-mutant glioma patient survival

Supplementary Material

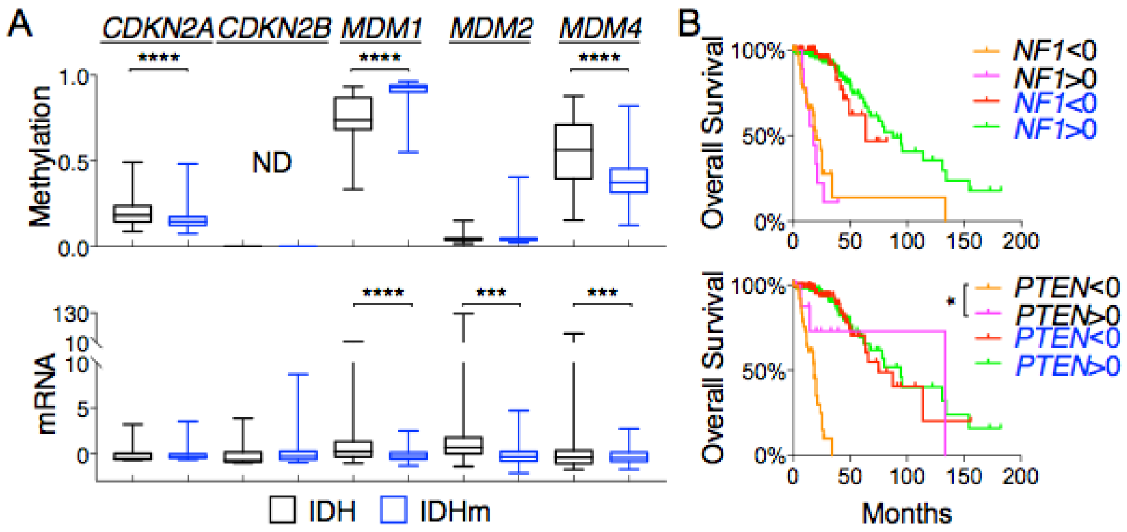

Supplementary Figure 1. Comparative analysis of TP53 and RB1 pathway genes and lack of survival benefits in IDH-mutant gliomas with respect to *NF1* and *PTEN* expression.

(A) DNA methylation and mRNA (in *z*-scores) of specified genes are presented in box-and-whisker plot for comparison of IDH-wildtype (IDH) and IDH-mutant (IDHm) groups. Unpaired *t*-tests were performed with two-tailed *p*-values indicated. ***, $p<0.001$; ****, $p<0.0001$; ND, not done. (B) Overall survival with respect to mRNA levels (in *z*-scores) of specified genes in IDH-wildtype gliomas (*in black*) and IDH-mutant gliomas (*in blue*) was analyzed separately in log-rank (Mantel-Cox) tests and presented with *p* values (unless not significant). *, $p<0.05$.

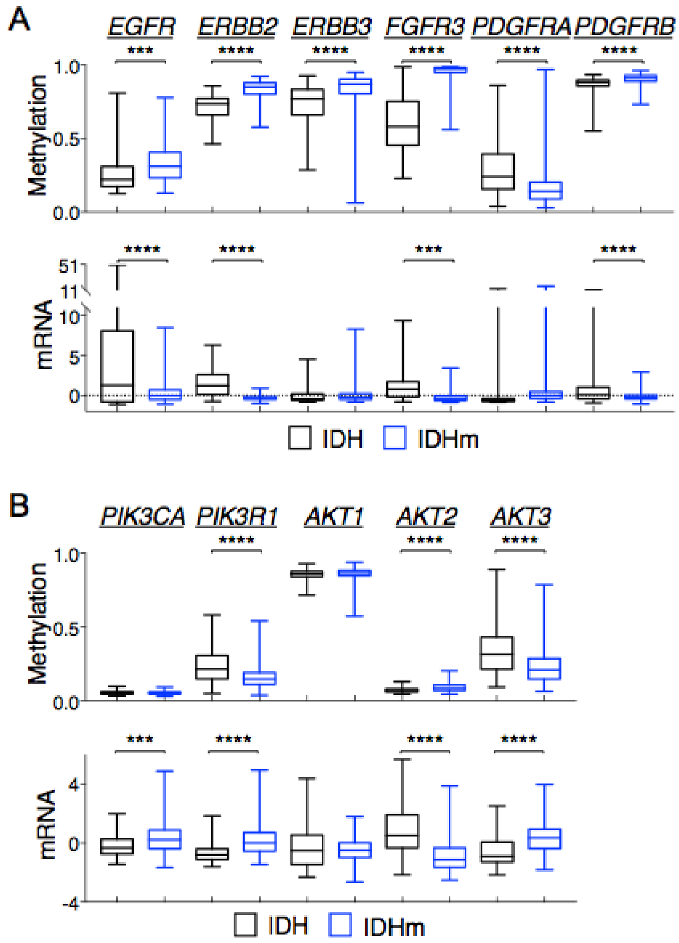

Supplementary Figure 2. Comparative analysis of genes in the RTK-PI3K-AKT pathway between IDH-wildtype and IDH-mutant gliomas.

(A and B) DNA methylation and mRNA (in *z*-scores) of RTK (*A*) and PI3K-AKT (*B*) genes are presented in box-and-whisker plots. Unpaired *t*-tests were performed for comparison between IDH and IDHm groups with two-tailed *p*-values indicated.

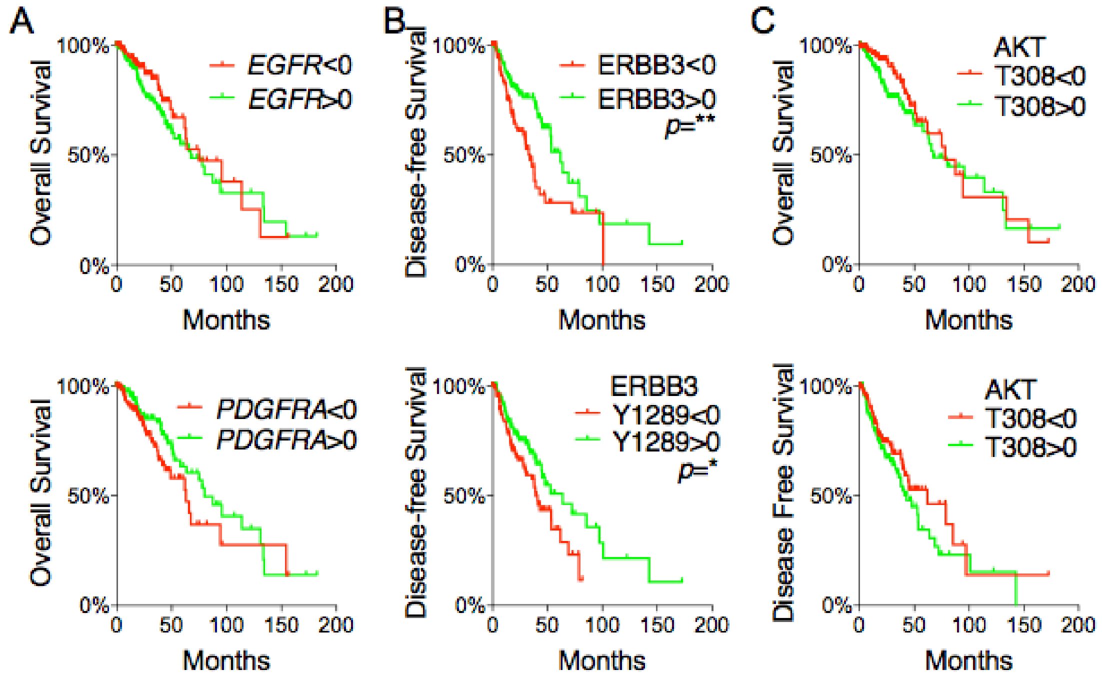

Supplementary Figure 3. Increased ERBB3 protein abundance and phosphorylation correlates with improved disease-free survival.

(A) Overall survival with respect to *EGFR* and *PDGFRA* mRNA levels, (B) disease-free survival with respect to ERBB3 abundance and phosphorylation, and (C) overall survival and disease-free survival with respect to AKT T308 phosphorylation were analyzed in log-rank (Mantel-Cox) tests. *p*-values are indicated (unless not significant).

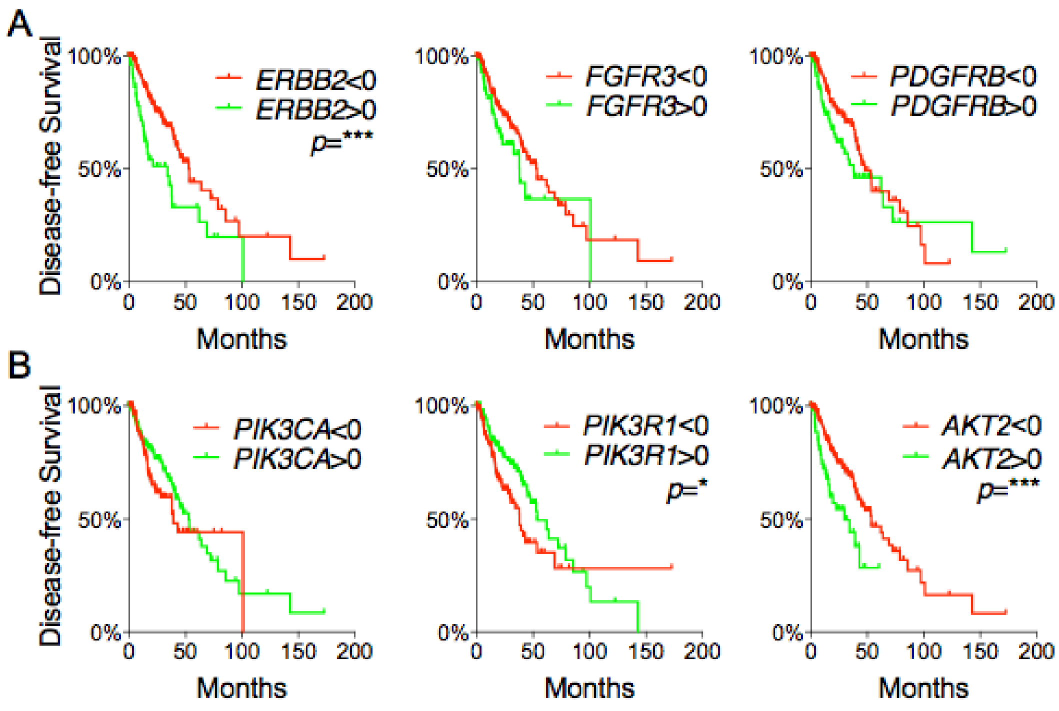

Supplementary Figure 4. Improved disease-free survival correlate with decreased expression of *ERBB2* and *AKT2* and increased expression of *PIK3R1*.

(A and B) Disease-free survival of glioma patients with respect to mRNA levels (in *z*-scores) of specified RTK genes (*A*) and PI3K–AKT genes (*B*) was analyzed in log-rank (Mantel-Cox) tests and presented with *p* values (unless not significant).

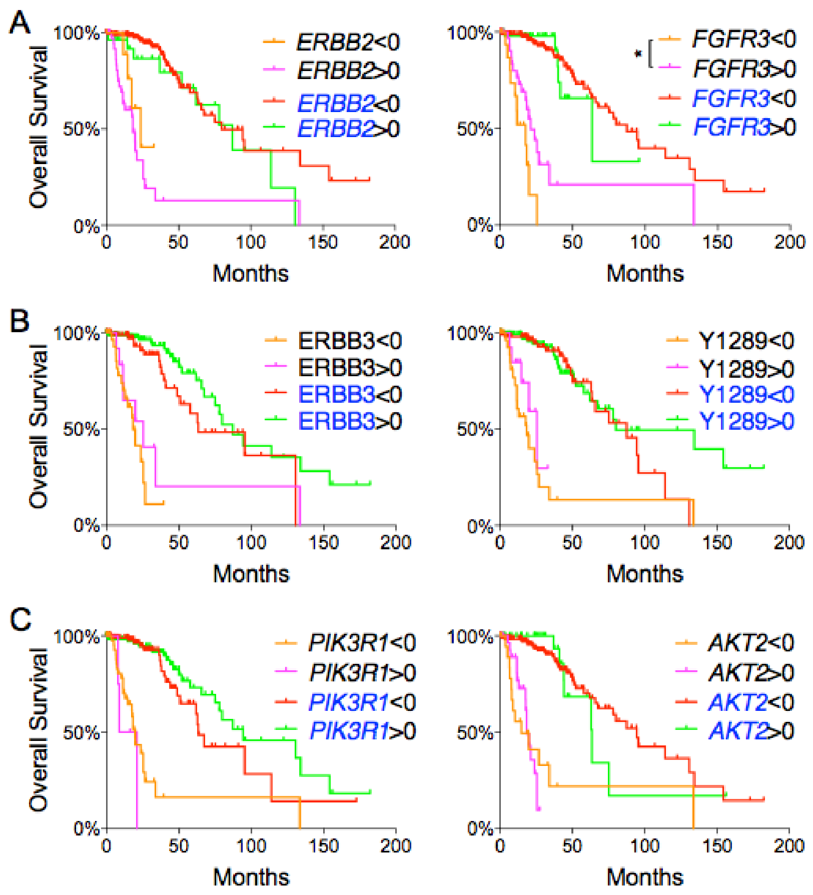

Supplementary Figure 5. Lack of prognostic values in IDH-mutant glioma with respect to genes of the RTK–PI3K–ATK pathway.

(A and C) Overall survival of patients in the IDH-wildtype group (*in black*) and IDH-mutant group (*in blue*) was analyzed separately in log-rank (Mantel-Cox) tests with respect to mRNA levels of *ERBB2*, *FGFR3* (*A*), *PIK3R1*, and *AKT2* (*C*), as specified. (B) Overall survival of patients in the IDH-wildtype group (*in black*) and IDH-mutant group (*in blue*) was analyzed separately in log-rank (Mantel-Cox) tests with respect to ERBB3 abundance and Y1289 phosphorylation.

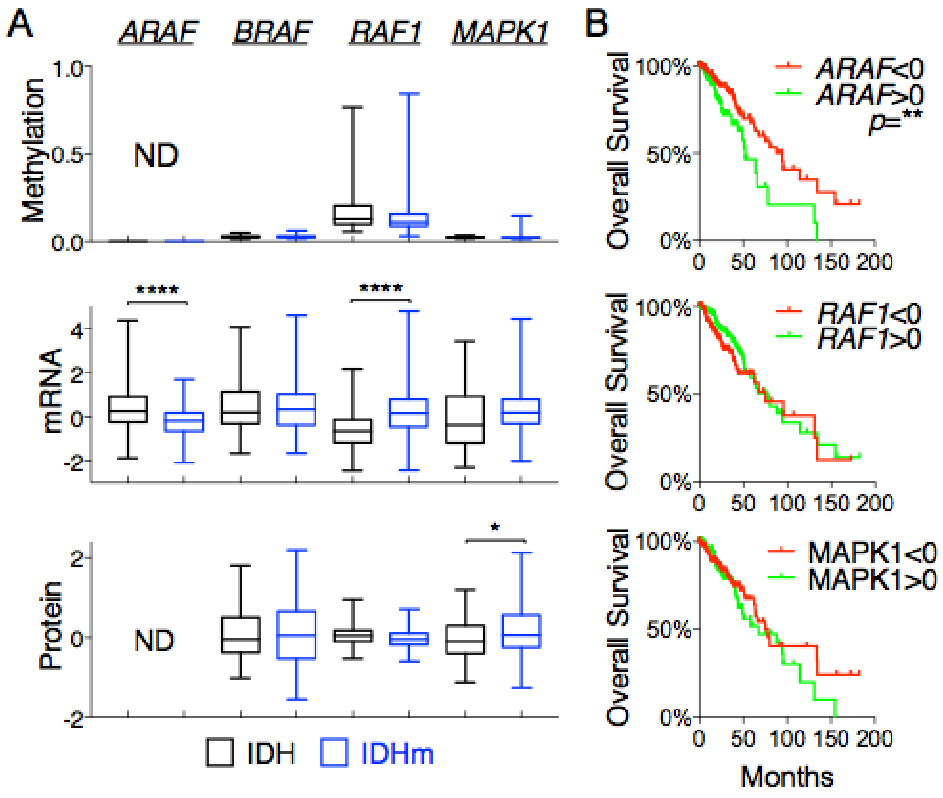

Supplementary Figure 6. The *ARAF* gene of the RAF-MAPK pathway correlates with overall survival of glioma patients.

(A) DNA methylation, mRNA (in *z*-scores), and protein abundance (in *z*-scores) of genes, as indicated, are presented in box-and-whisker plots. (B) Overall survival of glioma patients with respect to mRNA levels of specified genes was analyzed in log-rank (Mantel-Cox) tests and presented with *p* value (unless not significant).

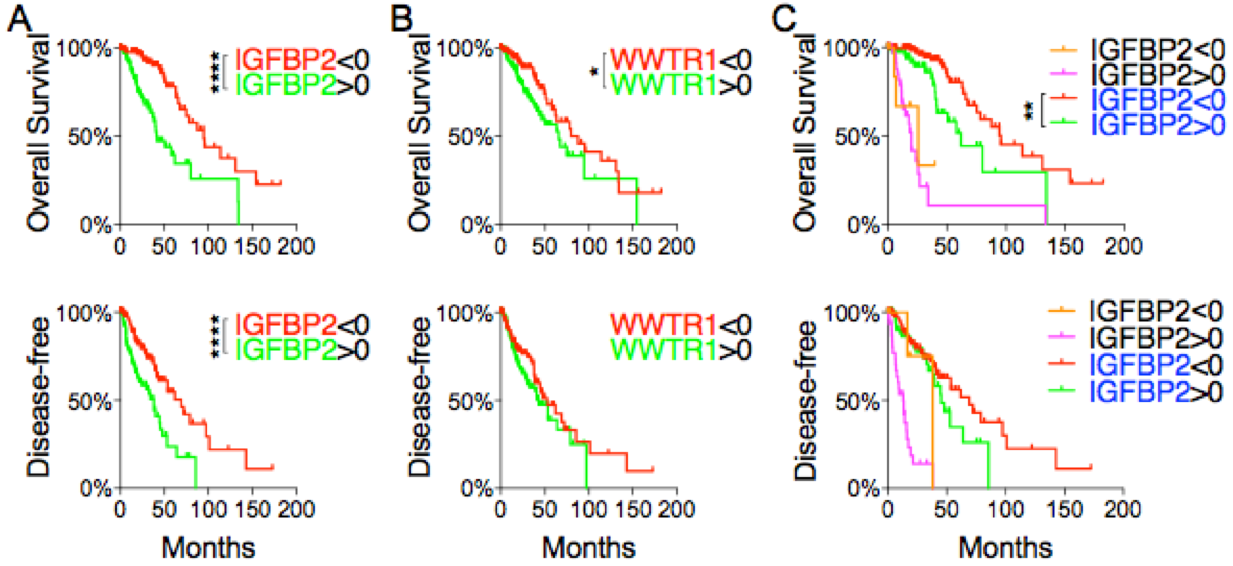

Supplementary Figure 7. *IGFBP2* protein abundance correlates with glioma patient survival and IDH-mutant patient survival.

(A and B) Overall and disease-free survival of glioma patients with respect to IGFBP2 (*A*) and WWTR1 (*B*) protein abundance was analyzed in log-rank (Mantel-Cox) tests. (C) Overall and disease-free survival of glioma patients in the IDH-wildtype group (*in black*) and IDH-mutant group (*in blue*) was analyzed separately in log-rank (Mantel-Cox) tests with respect to IGFBP2 protein abundance.

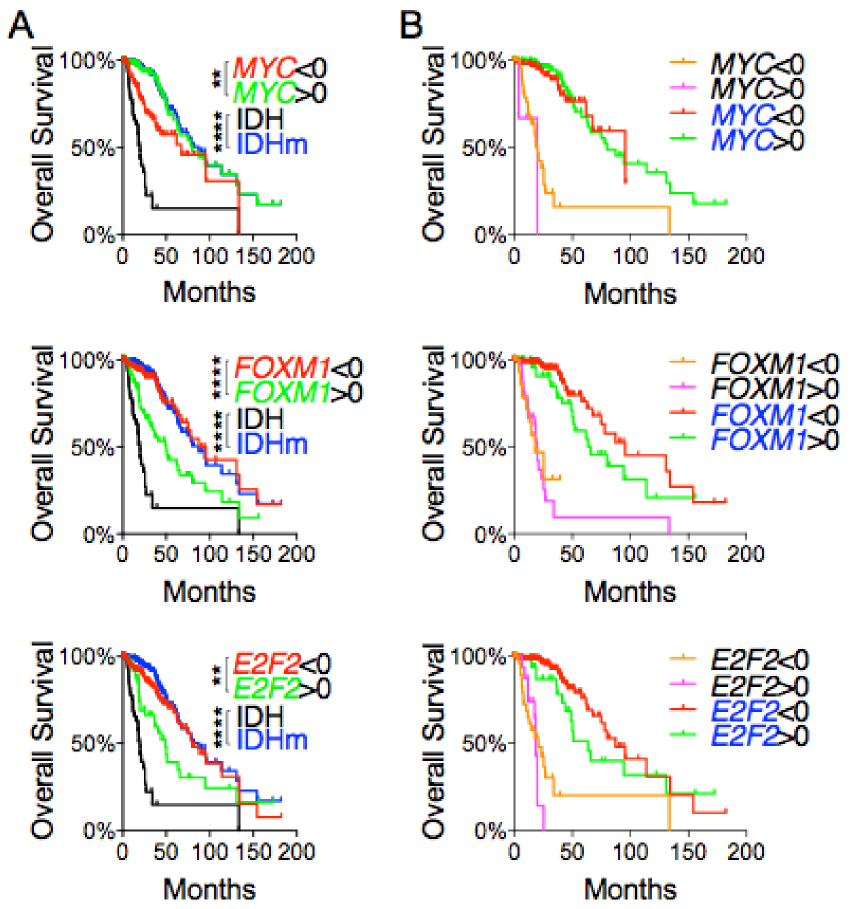

Supplementary Figure 8. Glioma progression genes *MYC1*, *FOXM1*, and *E2F2* are prognostic in glioma patients but not IDH-mutant glioma patients.

(A) Overall survival of glioma patients with respect to mRNA levels of *MYC1*, *FOXM1*, and *E2F2,* as specified, was analyzed in log-rank (Mantel-Cox) tests. (B) Overall survival of patients in the IDH-wildtype group (*in black*) and IDH-mutant group (*in blue*) was analyzed separately in log-rank (Mantel-Cox) tests with respect to *MYC1*, *FOXM1*, and *E2F2* mRNA levels.

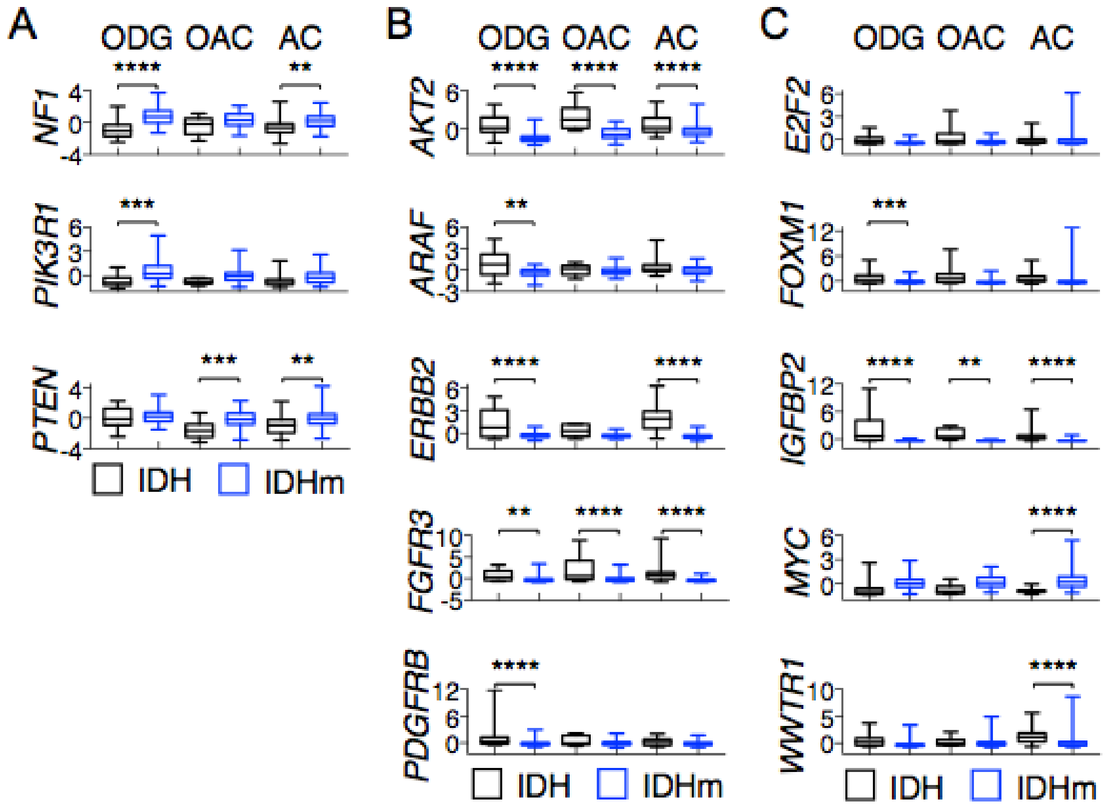

Supplementary Figure 9. Comparative analysis between IDH-wildtype and IDH-mutant oligodendroglioma, oligoastrocytoma, and astrocytoma with respective to gene expression associated with patient survival.

(A–C) Expression of tumor-suppressor genes (*A*), oncogenes (*B*), and glioma progression genes (*C*) was compared between IDH and IDHm groups in oligodendroglioma (ODG), oligoastrocytoma (OAC), and astrocytoma (AC). One-way ANOVA analysis was performed with *p*-values indicated (unless not significant).
