# Supplementary material for: IGFBP2 expression predicts IDH-mutant glioma patient survival
Source: Oncotarget. 2016 Nov 12;8(1):191–202. doi: 10.18632/oncotarget.13329 (PMC5352106; doi:10.18632/oncotarget.13329)
Supplement: Supplementary file 2 [file oncotarget-08-191-s002.pdf]

IGFBP2 expression predicts IDH-mutant glioma patient survival

Supplementary Material

Supplementary Table 1: Mutation Frequencies in Lower-grade Glioma

Gene	IDH Wildtype	IDH Mutant
NF1	21%	3%
PIK3CA	9%	8%
PIK3R1	2%	6%
PTEN	25%	0%
RB1	5%	0%
TP53	15%	59%

Supplementary Table 2: Overall Survival of Lower-grade Glioma Patients

Gene	Median Survival (months)	*p* value	Hazard Ratio (A/B)	95% CI of Ratio
***AKT2*<0**	87.39	0.0015	0.46	0.2522 to 0.8437
***AKT2*>0**	43.86			
***ARAF*<0**	94.45	0.0039	0.52	0.3179 to 0.8569
***ARAF*>0**	52.07			
***E2F2*<0**	79.93	0.0255	0.58	0.3384 to 1.003
***E2F2*>0**	48.98			
***ERBB2*<0**	79.93	<0.0001	0.29	0.1622 to 0.5018
***ERBB2*>0**	26.74			
**ERBB3<0**	57.88	0.0022	2.07	1.243 to 3.454
**ERBB3>0**	87.39			
**ERBB3-Y1289<0**	65.70	0.0144	1.82	1.117 to 2.967
**ERBB3-Y1289>0**	79.93			
***FGFR3*<0**	79.93	0.0047	0.51	0.2858 to 0.9024
***FGFR3*>0**	41.46			
***FOXM1*<0**	87.39	0.0001	0.41	0.2527 to 0.6703
***FOXM1*>0**	50.10			
***IGFBP2*<0**	87.39	<0.0001	0.11	0.0464 to 0.2624
***IGFBP2*>0**	17.67			
**IGFBP2<0**	94.45	<0.0001	0.32	0.1876 to 0.5314
**IGFBP2>0**	41.46			
**IDH**	19.88	<0.0001	7.20	3.153 to 16.42
**IDHm**	87.39			
**IDH-*IGFBP2*<0**	Undefined	0.0308	0.24	0.1021 to 0.5676
**IDH-*IGFBP2*>0**	18.36			
**IDHm-*IGFBP2*<0**	87.39	<0.0001	0.05	0.0009576 to 3.058
**IDHm-*IGFBP2*>0**	14.98			
**IDHm-IGFBP2<0**	95.50	0.0040	0.42	0.2077 to 0.857
**IDHm-IGFBP2>0**	61.96			

**IDH-*PTEN*<0**	18.36	0.0121	3.71	1.676 to 8.208
**IDH-*PTEN*>0**	133.60			
***MYC*<0**	67.41	0.0014	2.05	1.276 to 3.286
***MYC*>0**	79.93			
***NF1*<0**	48.98	0.0051	1.89	1.125 to 3.168
***NF1*>0**	79.93			
***PTEN*<0**	62.91	0.0032	1.89	1.191 to 3.006
***PTEN*>0**	94.45			
***PIK3R1*<0**	61.96	0.0006	2.24	1.405 to 3.562
***PIK3R1*>0**	94.45			
***RB1*<0**	134.20	0.0482	0.61	0.3849 to 0.9776
***RB1*>0**	67.41			
***WWTR1*<0**	79.93	0.0023	0.50	0.3034 to 0.8095
***WWTR1*>0**	62.91			
**WWTR1<0**	87.39	0.0241	0.59	0.3736 to 0.9448
**WWTR1>0**	65.70			

Comparison of RNA expression (genes in *italic* typeface), protein abundance and phosphorylation (roman typeface) between *z*-scores less than zero and greater than zero of individual genes. IDH, IDH-wildtype glioma patients; IDHm, IDH-mutant glioma patients.

Supplementary Table 3: Disease-free Survival of Lower-grade Glioma Patients

Gene	Median Survival (months)	*p* value	Hazard Ratio (A/B)	95% CI of Ratio
***AKT2*<0**	53.45	0.0010	0.48	0.2687 to 0.8425
***AKT2*>0**	29.66			
***ERBB2*<0**	53.45	0.0004	0.46	0.2683 to 0.7877
***ERBB2*>0**	34.00			
**ERBB3<0**	34.00	0.0019	1.92	1.231 to 2.981
**ERBB3>0**	61.96			
**ERBB3-Y1289<0**	39.32	0.0282	1.59	1.036 to 2.426
**ERBB3-Y1289>0**	63.83			
**IDH**	13.30	<0.0001	5.51	2.435 to 12.49
**IDHm**	53.52			
**IDH-*IGFBP2*<0**	37.68	0.0273	0.29	0.1259 to 0.6626
**IDH-*IGFBP2*>0**	9.43			
**IDHm-*IGFBP2*<0**	61.96	0.0068	0.18	0.006998 to 4.582
**IDHm-*IGFBP2*>0**	29.66			
***IGFBP2*<0**	53.52	<0.0001	0.14	0.05582 to 0.3605
***IGFBP2*>0**	9.43			
**IGFBP2<0**	68.89	<0.0001	0.44	0.2806 to 0.6963
**IGFBP2>0**	37.91			
***PIK3R1*<0**	37.91	0.0441	1.52	1 to 2.299
***PIK3R1*>0**	53.52			
***WWTR1*<0**	13.30	0.0071	0.57	0.3613 to 0.8952
***WWTR1*>0**	53.52			

Comparison of RNA expression (in *italic* typeface), protein abundance and phosphorylation (in roman typeface) between *z*-scores less than zero and greater than zero of individual genes. IDH, IDH-wildtype glioma patients; IDHm, IDH-mutant glioma patients.
